# Identification of salt stress-tolerant candidate genes in the BC_2_F_2_ population at the seedling stages of *G. hirsutum* and *G. darwinii* using NGS-based bulked segregant analysis

**DOI:** 10.3389/fpls.2023.1125805

**Published:** 2023-07-03

**Authors:** Muhammad Shehzad, Allah Ditta, Xiaoyan Cai, Shafeeq Ur Rahman, Yanchao Xu, Kunbo Wang, Zhongli Zhou, Liu Fang

**Affiliations:** ^1^ State Key Laboratory of Cotton Biology, Institute of Cotton Research, Chinese Academy of Agricultural Sciences, Anyang, Henan, China; ^2^ Plant Breeding and Genetics Division, Cotton Group, Nuclear Institute for Agriculture and Biology (NIAB), Faisalabad, Punjab, Pakistan; ^3^ National Nanfan Research Institute of Chinese Academy of Agriculture Sciences, Sanya, China; ^4^ MOE Laboratory for Earth Surface Processes, College of Urban and Environmental Sciences, Peking University, Beijing, China; ^5^ School of Agricultural Sciences, Zhengzhou University, Zhengzhou, Henan, China

**Keywords:** bulked segregant analysis, salt stress, candidate gene, cotton, polymorphic markers

## Abstract

Salinity is a major threat to the yield and productivity of cotton seedlings. In the present study, we developed a BC_2_F_2_ population of cotton plants from *Gossypium darwinii* (5–7) and *Gossypium hirsutum* (CCRI 12–4) salt-susceptible parents to identify salt-resistant candidate genes. The Illumina HiSeq™ strategy was used with bulked segregant analysis. Salt-resistant and salt-susceptible DNA bulks were pooled by using 30 plants from a BC_2_F_2_ population. Next-generation sequencing (NGS) technology was used for the sequencing of parents and both bulks. Four significant genomic regions were identified: the first genomic region was located on chromosome 18 (1.86 Mb), the second and third genomic regions were on chromosome 25 (1.06 Mb and 1.94 Mb, respectively), and the fourth was on chromosome 8 (1.41 Mb). The reads of bulk1 and bulk2 were aligned to the *G. darwinii* and *G. hirsutum* genomes, respectively, leading to the identification of 20,664,007 single-nucleotide polymorphisms (SNPs) and insertions/deletions (indels). After the screening, 6,573 polymorphic markers were obtained after filtration of the candidate regions. The SNP indices in resistant and susceptible bulks and Δ(SNP-index) values of resistant and susceptible bulks were measured. Based on the higher Δ(SNP-index) value, six effective polymorphic SNPs were selected in a different chromosome. Six effective SNPs were linked to five candidate genes in four genomic regions. Further validation of these five candidate genes was carried out using reverse transcription-quantitative polymerase chain reaction (RT-qPCR), resulting in an expression profile that showed two highly upregulated genes in the salt-tolerant species *G. darwinii*, i.e., *Gohir.D05G367800* and *Gohir.D12G239100*; however, the opposite was shown in *G. hirsutum*, for which all genes, except one, showed partial expression. The results indicated that *Gohir.D05G367800* and *Gohir.D12G239100* may be salt-tolerant genes. We are confident that this study could be helpful for the cloning, transformation, and development of salt-resistant cotton varieties.

## 1 Introduction

Cotton has long been a major source of fiber and is an essential raw material that is used in textile industries all around the world (Renny-Byfield et al., 2016). However, in previous years, the practices of inbreeding and rigorous selection for superior morphological traits have adversely affected cotton’s diversity, decreasing the current genetic diversity of cotton varieties (Abdurakhmonov et al., 2008). Cotton is a mesophytic plant; hence, its yield and fiber quality is severely affected by biotic and abiotic stresses (Dabbert and Gore, 2014). Out of all abiotic stresses, salinity most affects cotton physiology and morphology. Salinity inhibits primary root development (Reinhardt and Rost, 1995), suppresses root length, and secondary root growth (Leidi, 1994), and decreases both the number of bolls and the yield (Uma and Patil, 1996). Salinity stress may also inhibit nutrient uptake and alter the function of photosynthetic mechanisms (Zhu, 2001). Because of the limited gene pool of today’s superior cotton types, developing stress-resistant cotton cultivars has become difficult (Zhang et al., 2014). Cotton is a salt-tolerant crop, but the rapid increase of salt-affected areas and the decline in levels of water available for agricultural land still necessitate superior cotton genotypes that perform better in the field, are more water-efficient, and are capable of high yields in saline soil (Peng et al., 2014).

As salt stress is worsening the situation, the solution to this problem is to integrate conventional breeding with modern genomic techniques to identify the genomic regions or genes that show resistance to or tolerance to salt stress. Complex cotton characteristics have been evaluated via association mapping and biparental mapping. Few studies have reported on trait mapping in which researchers were focused on abiotic stresses, such as important phenotypic traits linked to salt stress in cotton crops. Efforts to investigate various abiotic stresses, such as salinity and drought tolerance, with the main focus being on understanding the manifestations of physiological and agronomic features with the help of next-generation sequencing (NGS) technologies, have been made (Vadez et al., 2012; Hamwieh et al., 2013).

Recent advancements in NGS have brought about a rapid decline in sequencing costs, making it possible to apply genomics for crop development in a cost-effective manner (Varshney et al., 2014). The benefit of identifying large numbers of single-nucleotide polymorphisms (SNPs) and insertions and deletions (indels) by resequencing has made it possible to detect and refine candidate genomic regions more effectively compared with old mapping methods such as quantitative trait locus (QTL) analysis (Chen et al., 2014). Identification of a gene or locus of a trait is one of the main tools to characterize gene function, which is ultimately used for the development of agronomic traits in crops (Takeda and Matsuoka, 2008). Bulk segregant analysis (BSA) is a fast technology used for identifying markers linked with traits of interest and, after being integrated with NGS technology, has reduced time and led to the swift identification of candidate genomic regions (Michelmore et al., 1991). BSA analysis has been utilized in various organisms to map vital genes (Whipple et al., 2011). This method has been applied to a wide variety of plants, including *Arabidopsis thaliana* (Schneeberger et al., 2009), rice (Abe et al., 2012; Wambugu et al., 2018), soybean (Chen et al., 2014), wheat (Trick et al., 2012), pigeon pea (Singh et al., 2016), sunflower (Livaja et al., 2013), and sorghum (Han et al., 2015), and has identified certain QTLs and candidate genes for key traits. It has been reported that the *AtNHX1* gene is involved in the Na^+^/H^+^ antiporter mechanism, which enhances sodium uptake into vacuoles and improves salt tolerance and plant biomass under salt stress conditions in *A. thaliana* (He et al., 2005). In BSA technology, two opposite pools are constructed by mixing the DNA of individuals with the highest and lowest trait values in the segregating population (Takagi et al., 2013). By analyzing the differences in SNPs and indels between the two pools, we can quickly locate the molecular markers that are strongly connected to desirable genes (Klein et al., 2018).

Sequencing genomes via NGS technologies integrated with BSA is a novel method for speeding up the detection of candidate genes that control key agronomic traits in many crops (Takagi et al., 2013; Lu et al., 2015). Li et al. (2015) sequenced the genome of cultivated upland cotton (*Gossypium hirsutum* TM-1); the sequenced data included 70,478 protein-coding genes. Wang et al. (2012) sequenced the entire diploid D-genome of *Gossypium raimondii*, which contained 37,505 genes (Wang et al., 2012). Liu et al. (2015) sequenced the extra-long fiber (AD)_2_ genome of *Gossypium barbadense*, which contained 77,358 genes (Liu X. et al., 2015). Li et al. (2014) sequenced the genome of *Gossypium arboretum*, which contained 41,331 genes and represents a main source for plant scientists, mainly those interested in the genetic development of cotton crops. The utilization of wild cotton progenitors is important to mitigating the effects of many abiotic and biotic stresses in plants (Kirungu et al., 2018). Several studies have been reported on F_2_ populations developed from two tetraploid cotton genotypes, but few studies have reported on backcross populations. The backcross technique enables the high-level maintenance of recurrent parent genomic content and a high-level of control of the genetic materials passed from the donor parents (Hospital, 2005). In addition, the backcrossing technique has been used to increase the genetic base of cucumber (*Cucumis sativus* L.) (Behera et al., 2011). According to previous research, backcrossing is an effective method for improving the performance of crops; we used a recurrent parent (*G. hirsutum)* and a donor parent (*G. darwinii)* for the development of a backcross population. The BC_2_F_2_ populations were genotyped using third-generation BSA technology. In order to identify candidate genes for salt tolerance, these genes were introgressed from a wild parent into a recurrent parent. The objective of this study was to discover candidate genes that confer salt stress-tolerance to cotton plants at the seedling stage. The Illumina HiSeq™ platform was used on a BC_2_F_2_ population, crossed between a salt-tolerant parent [i.e., *G. darwinii* (5–7)] and a salt-susceptible parent [i.e., *G. hirsutum* (CCRI 12–4)]. The Δ(SNP-index) procedure was used for the identification of associated genomic regions. This study will be helpful in expanding our understanding of the mechanisms underlying salt tolerance in cotton and will also provide a basis for future studies to build upon.

## 2 Materials and methods

### 2.1 Experimental site

This research was carried out under controlled greenhouse conditions at the Institute of Cotton Research, the Chinese Academy of Agricultural Sciences, Anyang, China.

### 2.2 Plant material and development of the mapping population

Backcross inbred lines were developed from *G. hirsutum* (CCRI 12–4), that is, the recurrent parent, and wild *G. darwinii* (5–7), that is, the donor parent. The recurrent parent *G. hirsutum* (CCRI 12–4) variety is cultivated in more than 90% of the area of China. Furthermore, its production is lower because it is less tolerant of salt stress. The CCRI 12–4 variety was developed by the Institute of Cotton Research, Chinese Academy of Agricultural Sciences, Anyang, Henan Province, China (Magwanga et al., 2018). Wild *G. darwinii* was used as a donor parent. This species of plant emerged from the Galápagos Islands. In addition, it closely resembles *G. barbadense* and has excellent characteristics, such as fiber fineness, salinity, drought tolerance, and resistance to *Verticillium* wilts and *Fusarium* diseases (Chen et al., 2015; Shehzad et al., 2019). *G. hirsutum* (CCRI 12–4) was crossed with the wild parent *G. darwinii* (5–7), which resulted in the F_1_ being crossed again with a recurrent parent, with *G. hirsutum* being used to develop the BC_1_F_1_ generation. The BC_1_F_1_ generation was crossed with the recurrent parent a second time, which generated BC_2_F_1_. The resulting BC_2_F_1_ was selfed and BC_2_F_2_ populations developed. In total, 410 lines were developed. Two hundred backcross progenies were used in this study due to insufficient seeds being available.

### 2.3 Hydroponic trials

Two hundred backcross BC_2_F_2_ progenies were grown for screening of resistance lines and parents under 150 mmol sodium chloride stress in hydroponic culture. The 200 BC_2_F_2_ lines with parents were assessed when grown under two salt concentrations of stress in a hydroponic system: one was the control treatment [0 mmol sodium chloride (NaCl)], and the second was the study treatment (150 mmol NaCl). The hydroponic culture chosen for this research was the same as that reported by Oluoch et al. (2016), with three biological repeats. Ten good seeds from each BC_2_F_2_ line and parent were selected after seed grading and spread on triple-layer filter paper in a vertical column for germination. Approximately 100 mL of distilled water was then added to the container and fixed in an incubator at 31°C for 72 h. In each line, three replicates were maintained under optimal growth conditions. The adopted experimental design was a completely randomized block design (CRBD). Three same-size seedlings for each replication were chosen and shifted to holes in Thermopore^®^ sheets, where each seedling was fixed with a soft sponge. Each container contained seven liters of half-strength Hoagland nutrient solution (Hoagland and Arnon, 1950). Air pumps were fixed in solution to supply oxygen to the root for proper growth. The greenhouse temperature was adjusted between 27°C and 29°C with a photoperiod of 14 hours of light/10 hours of dark. At the emergence of the third leaves, a 25 mmol NaCl solution was added each day to the solution until a final concentration of 150 mM was attained; no NaCl solution was added to plants grown under control conditions. After 1 week, the solution was changed and the containers were rearranged. The growth performance of the BC_2_F_2_ population and their parental lines were assessed for salt tolerance in terms of seven morphological traits. After 2 weeks of seedling growth in 150 mmol NaCl, data were collected. A leaf chlorophyll meter was used for the measurement of chlorophyll content (CHL) (SPAD 502 meter; Minolta Osaka, Japan). The CHL content data were obtained based on the average data for the bottom leaf, middle leaf, and top fresh leaf from each plant. The roots and shoots of each plant were harvested individually and cleaned with distilled water. Shoot fresh weight (SFW), shoot length (SL), root length (RL), and root fresh weight (RFW) were measured. Fresh leaf and root tissue were placed in the oven at a temperature of 80°C and, after 48 h, root dry weight (RDW) and shoot dry weight (SDW) data were collected. Pearson correlation analysis (PCA) and the phenotypical variation among these traits were measured using SPSS software (version 16.0; IBM Corporation, Armonk, NY, USA).

### 2.4 Construction of sample pools

Resistant and susceptible bulks were prepared based on the phenotypic data obtained from the three replicates (i.e., R1, R2, and R3) grown under salt-stress conditions. The 30 resistant BC_2_F_2_ lines were selected from 200 BC_2_F_2_ lines based on the highest mean values of phenotypic data and found to have three traits common, that is similar values for SFW, SDW, and RFW. Thirty susceptible BC_2_F_2_ lines were selected, with lower mean values occurring for these three traits. Young leaves from parents, resistant lines, and susceptible line samples were collected and instantly frozen in liquid nitrogen before being stored at –80°C for DNA extraction. The Takara MiniBEST Plant Genomic Kit (TAKARA BIO Inc., Beijing, China) was used to extract DNA.

### 2.5 Library construction and resequencing

A total of four Illumina libraries were prepared, from two parents, 30 resistant progenies, and 30 susceptible progenies (P1, P2, bulk1, and bulk2). Subsequently, the genomic DNA sample was tested for DNA quality using the NanoDrop spectrophotometer. For quality control of sequences (QC-seq) analysis, resistant and susceptible bulks DNA pools were developed from the BC_2_F_2_ population after mixing the same quantity of both DNA pools separately. The samples of DNA were sonicated to build 400-bp fragments, which were then repaired at the 3′ ends, connected to an “A” tail, and ligated to the complete adapter for Illumina high-throughput sequencing via PCR amplification. In addition, the final PCR products were purified with the AM-Pure XP system. The Agilent 2100 Bioanalyzer was used for the size division of the libraries. The quantification of the libraries was carried out using reverse transcription-quantitative PCR (RT-qPCR). The products were sequenced with the help of Illumina Hi-Seq 4000 technology to build 150-bp paired-end reads with an insert size of approximately 350 bp. After sequencing, small numbers of poor-quality primary Illumina data or raw data remain; consequently, low-quality reads were filtered in accordance with the following quality control procedure.

Step 1: The adapter sequence from the reads was removed.
Step 2: The 5′ end was cut off if the sequencing quality value was below 20 or had been identified as the “N” base.
Step 3: The 3′ end was cut off if the sequencing quality value was below three or identified as the “N” base.
Step 4: four bases were used for the window; the base was cut off if the window had an average mass value of less than 20.
Step 5: The reads containing “N” with a rate of 10% were removed.
Step 6: We cut the base mass value of more than 40% below 15 reads.
Step 7: we discarded adapters and a length of less than 30 bp after mass pruning.

The quality inspection qualified the library to use the Illumina HiSeq platform to carry out the sequencing. The sequencing strategy used was Illumina PE150, and the total sequencing read length was 350 bp.

### 2.6 Assembly and calculation of the SNP index

After using Illumina HiSeq sequencing, raw data were obtained. The clean data were generated through the removal of low-quality reads from the raw data. The process for this was as follows: first, the Burrows–Wheeler aligner (BWA) software (Li and Durbin, 2009) was used to compare the sequencing data with the *Gossypium* genome sequence; the position attribution of the sequence stored as a binary alignment map (BAM) file, on which SNP tests and small Indels tests were carried out. Second, the Samtool software (Li et al., 2009) was use to delete duplicate sequences. Third, the genotypic differences between each sample and the *Gossypium* genome sequence were analyzed using the haplotype algorithm of the genome analysis toolkit (GATK) software (McKenna et al., 2010). The analysis of different samples was combined, and the difference in data between the products was determined. The resistant parent’s variants were then used to construct the reference-guided assembly of the resistant parent, *G. darwinii*, by replacing the bases in the genome with confidence variant calls. After the alignment of resistant and susceptible poles, reads with previously formed assembly variations were located in both poles.

We targeted the mutation sites and annotation of the genes with the help of the formula described by Abe et al. (2012) and found them around the mutant loci.


(1) SNP index=DepM/(DepM+DepW)

(2) ΔSNP index value=Index(Mut)−Index(Wild)

The mutant parent index was measured as the depth of the mutant plus the depth of the wild divided by the depth of the mutant. A similar procedure was applied to the index of a wild parent. The Δ index values were determined by subtracting the mutant index from the wild parent index. If the genome of the bulked DNA was related to the resistant parent (*G. darwinii*), it meant that the Δ index was equal to one. If the Δ index was equal to one under this condition, the genome of bulked DNA was consistent with the susceptible parent and the Δ index was equal to zero, which meant that both parents contributed equally. If the Δ index was equal to –1, it meant that the alleles came from resistant parents. Sniff software (Cingolani et al., 2012) was used for functional annotation of the identified genes.

### 2.7 RT-qPCR validation of the candidate genes for salt tolerance in *G. darwinii*, with *G. hirsutum* as a reference

*G. hirsutum* (CCRI 12–4) and *G. darwinii* 5-7 species were used in this research to investigate gene expression under 200 mmol salt treatment at three leaf stages while grown in a hydroponic system. Leaf samples were taken after 0 h, 1 h, 3 h, 6 h, and 24 h of salt stress treatment and quickly transferred into liquid nitrogen and stored at –80°C for RNA extraction. Under controlled conditions, seedlings were treated with only fresh water. The RNAprep Pure Plant Kit (Tianjin, China) was used for the extraction of RNA from leaf samples. The RNAprep Pure Plant Kit was used for the extraction of RNA from leaf samples. Single-stranded complementary DNA (cDNA) was synthesized using the RT reagent Takara kit, China. Protein kinase gene primers were designed from the online NCBI database. **Supplementary Table S6** contains primer information. RT-qPCR was carried out in accordance with the method described by Wang et al. (2020).

## 3 Results

### 3.1 Phenotypic identification under salt stress

**Figure 1:**
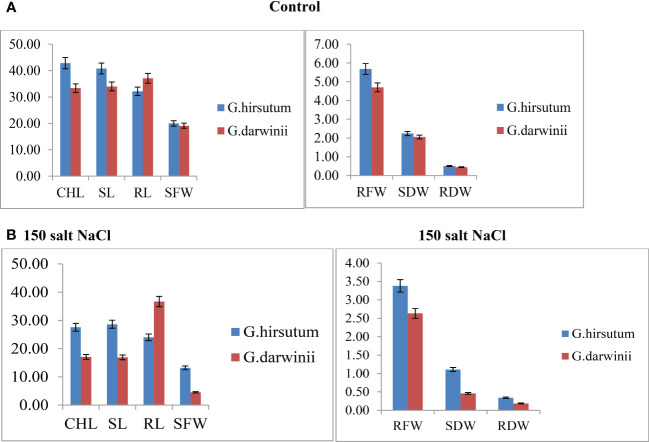
**(A, B).** Control condition of parents with seven morphological traits indicated by **(A)** and treated conditions under 150 mmol NaCl of parents with seven morphological traits indicated by **(B)**. The *x*-axis shows the number of traits, and the *y*-axis indicates the mean values of traits. CHL, chlorophyll content; RDW, root dry weight; RFW, root fresh weight; RL, root length; SDW, shoot dry weight; SFW, shoot fresh weight; SL, shoot length.

Both parents’ mean values of seven morphological traits were compared under control and stress conditions (**Figure 1**). Significant differences were observed between the parents under controlled conditions regarding CHL content, SL, RL, RDW, and RFW, whereas no difference was observed in SFW and SDW. Under control conditions, CCRI 12–4 had higher values in all traits except RL. In the treated condition, at 150 mmol NaCl, the CHL content of *G. darwinii* decreased in SPAD value to 18 (from 31), and in CCRI12, it decreased to 27 (from 46). The CHL content of *G. darwinii* showed a less significant decrease than that of CCRI 12–4. *G. darwinii* showed tolerance in two morphological trait values: RL, increased greatly, and CHL, which decreased to a lesser extent.

BC_2_F_2_ populations had a wide range of phenotypic variation in seven traits (CHL content, SL, RL, SFW, RFW, SDW, and RDW) under salt stress conditions (**Table 1**). The frequency distributions of all traits were found to be normal (**Supplementary Figure G1**). Correlation analysis was carried out using the mean values of four seedlings per replication for the 150 mmol salt treatment. All traits had a significantly positive correlation with each other except for RL/SL CHL content/SL, and CHL content/SDW, which showed no significant correlation. The maximum significant correlation was noted between SFW and SDW (0.927) under salt stress conditions, thus showing an association between these traits. For example, RFW was highly correlated with RL (0.259). The SDW had a significant correlation with RFW (0.764). The correlation between SDW and RDW was positively significant (0.812) (Tiwari et al., 2013). The CHL content under 150 mmol NaCl was significantly correlated with RFW (0.161) and SDW (0.122). Under control conditions, CHL content was highly significantly correlated with RDW (0.221) (**Table 2**).

**Table 1:** Phenotypic variation measured in seven traits in the BC_2_ F_2_ generation.

Trait	Units	ENV	Mean	Minimum	Maximum	SD	Skewness	Kurtosis
SL	Centimeters (cm)	N	24.62	12.68	34.39	4.05	–0.38	0.07
		T	21.31	11.01	29.10	3.46	–0.36	0.05
RL	Centimeters (cm)	N	29.63	19.48	50.60	4.49	0.57	1.52
		T	26.94	17.97	46.00	4.03	0.60	1.59
SFW	Grams (g)	N	8.83	1.68	23.77	4.40	0.72	0.34
		T	7.35	1.40	20.13	3.67	0.74	0.40
RFW	Grams (g)	N	4.06	0.65	11.81	2.03	0.85	1.14
		T	3.42	0.55	10.02	1.71	0.89	1.16
SDW	Grams (g)	N	0.97	0.19	2.49	0.51	0.64	–0.14
		T	0.88	0.17	2.21	0.46	0.65	–0.13
RDW	Grams (g)	N	0.30	0.02	0.77	0.15	0.45	0.32
		T	0.25	0.02	0.65	0.12	0.42	0.27
CHL	SPAD value	N	41.45	21.99	65.95	7.32	0.49	0.83
		T	36.75	19.09	57.97	6.28	0.43	0.99

CHL, chlorophyll content; RDW, root dry weight; RFW, root fresh weight; RL, root length; SDW, shoot dry weight; SFW, shoot fresh weight; SL, shoot length.

**Table 2:** PCA in seven morphological traits.

Trait	ENV	SL	RL	SFW	RFW	SDW	RDW	CHL
SL		1						
RL	N	0.103						
	T	0.09	1					
SFW	N	0.634**	0.274**					
	T	0.641**	0.272**	1				
RFW	N	0.596**	0.261**	0.723**				
	T	0.598**	0.259**	0.723**	1			
SDW	N	0.677**	0.253**	0.927**	0.764**			
	T	0.681**	0.244**	0.929**	0.765**	1		
RDW	N	0.556**	0.358**	0.793**	0.868**	0.813**		
	T	0.544**	0.370**	0.793**	0.867**	0.812**	1	
CHL	N	0.043	0.164*	0.155*	0.161*	0.114	0.221**	
	T	0.052	0.183**	0.165**	0.161*	0.122*	0.230**	1

**, Correlation is significant at the 0.01 level (one-tailed). * Correlation is significant at the 0.05 level (one-tailed). Control conditions are indicated by “N”, and treatment conditions are indicated by “T”.

CHL, chlorophyll content; RDW, root dry weight; RFW, root fresh weight; RL, root length; SDW, shoot dry weight; SFW, shoot fresh weight; SL, shoot length.

### 3.2 Construction of the BSA pools

Based on the phenotypic data collected from the CCRI 12–4 × *G. darwinii* BC_2_F_2_ population, two bulks, one resistant and one susceptible, were prepared for BSA analysis. The significant correlation between SFW and SDW (0.927) was under salt stress conditions, showing that there was an association between these traits. The SDW was highly correlated with RFW (0.764). The correlation between SFW and RFW was positively significant (0.723). The 30 resistant BC_2_F_2_ lines were selected on the basis of the three common traits, including mean values for SFW (10.87–19.79), SDW (1.30–2.13), and RFW (4.36–8.80). The same procedure was used for 30 susceptible BC_2_F_2_ lines, including SFW (1.55–4.26), SDW (0.17–0.50), and RFW (0.55–2.26).

### 3.3 Sequencing base content and error rate distribution

**Figure 2:**
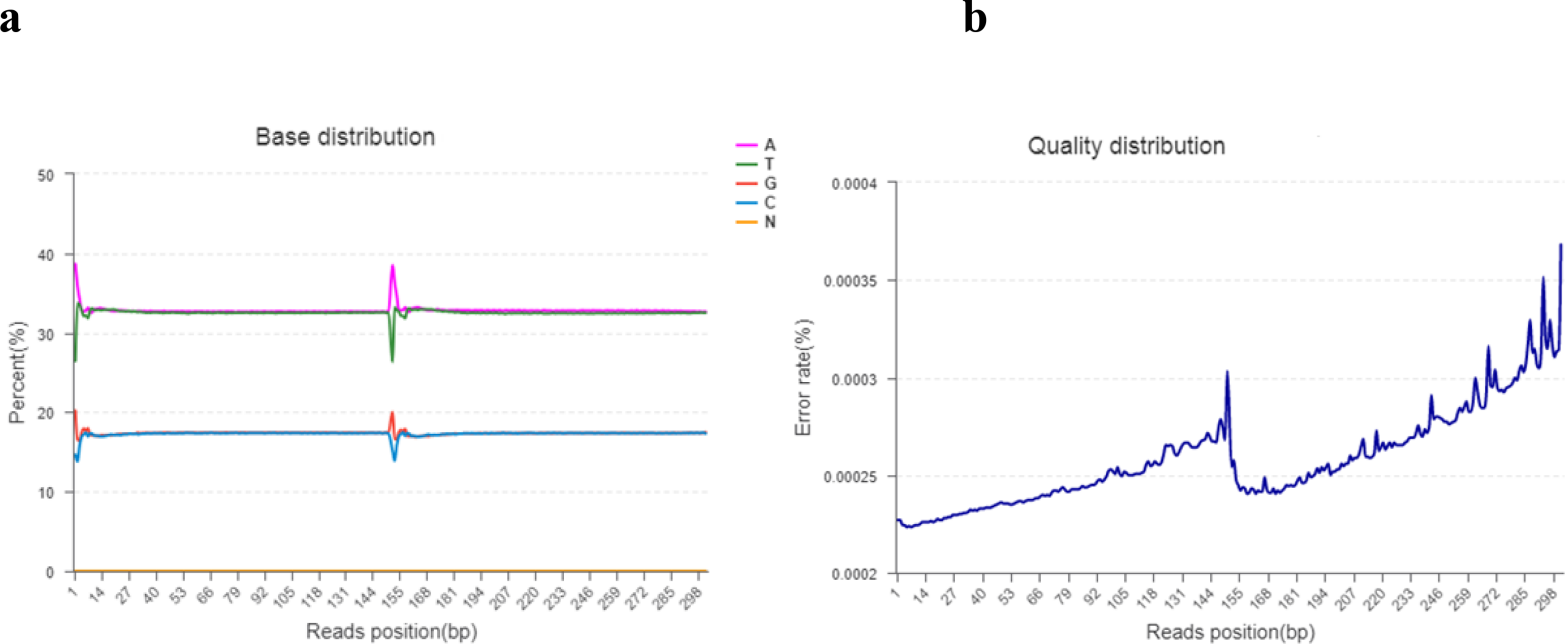
**(A, B). (A)** The horizontal line indicates the base of clean reads from the 5′ end to the 3′ end. In addition, the longitudinal line indicates the percentage of all clean data reads. Each base is indicated by a different color. **(B)** The horizontal line indicates the base pair position of the reads and the longitudinal line indicates the percentage of the base error rate of the entire reads.

Base content grouping examination is commonly used to find the distribution of adenine (A), thymine (T), guanine (g), and cytosine (C). AT and GC gave uncertainty of sequences and complementary bases. Theoretically, the GC and AT content is equal in each sequencing cycle, and the entire sequencing process is stable and unchanged. The “N” indicates that the sequencer cannot determine the less unknown protein bases. The sequencing error rate increases as the sequence length increases, which is due to the utilization of chemical reagents in the sequencing test. Furthermore, owing to the technical features of Illumina HiseqTM sequencing, the error rate at the end of sequencing fragments and the end of numerous cycles was high, as shown in **Figures 2A, B**.

### 3.4 Whole-genome resequencing and mapping of reads

Four Illumina libraries [one for the resistant bulk, one for the susceptible bulk, and two for the parents (i.e., CCRI 12–4 and *G. darwinii* 5–7)] were assembled and used in the Illumina PE150 sequencing strategy for whole-genome sequencing. In total, 291,637,437 raw reads for the resistant bulk, 347,206,384 raw reads for the susceptible bulk, 110,926,703 raw reads for CCRI 12–4, and 143,954,134 raw reads for *G. darwinii* were generated. After filtration, 291,626,020 clean reads for bulk1, 347,192,723 clean reads for bulk2, 110,919,876 for CCRI 12–4, and 141,174,760 clean reads for *G. darwinii* were produced. BWA software was used to generate mapped clean reads from CCRI 12–4 to the reference genome sequence obtained from the cotton genome (http://mascotton.njau.edu.cn). After alignment, the average depth of CCRI 12–4 was 10.61, and its genome coverage was 93.41. The average depth of *G. darwinii* was 28.78, and its genome coverage was 88.53. After the development of reference-based assemblies, CCRI 12–4 and *G. darwinii* were aligned with clean reads of resistant and susceptible bulks, resulting in average depths of 27.62 and 33.18 and genome coverages of 94.02 and 95.05 for the resistant and susceptible bulks, respectively. The clean guanine–cytosine (GC) percentage of sequences for both parents was 34.65% for CCRI 12–4, 35.77% for *G. darwinii*, 34.7% for bulk1, and 34.63% for bulk2. The clean Q30 score for CCRI 12–4, *G. darwinii*, bulk1, and bulk2 was 93.8%, 93.52%, 93.8%, and 93.52%, respectively, and showed that this sequencing method had a minimum error rate (**Table S1**).

One of the most polymorphic legacies on the genome is the SNP, which primarily refers to a DNA sequence polymorphism created by the alteration of a single nucleotide at the genomic level. SNP variants are divided into two types: conversions and transversions. Mutations between the same type of base (purine to purine, pyrimidine to pyrimidine) are called conversions (transition), and mutations between diverse forms of bases (purine and pyrimidine) are called transversions. In general, conversions are more likely to occur than transversions (**Table 3**). GATK technology (available online: https://software.broadinstitute.org/) was used to process the result (i.e., the BAM file) via the GATK haplotype procedure for the detection of SNPs.

**Table 3:** The details of the SNPs that were identified in two parents and two bulks.

Sample ID	SNP no.	Transition	Transversion	Ti/Tv	Heterozygosity number	Homozygosity number
*G. hirsutum* (CCRI 12–4)	2,208,798	1,509,021	699,275	2.16	1,556,770	652,028
*G. darwinii* (5–7)	16,936,679	11,647,053	5,266,567	2.21	4,386,035	12,550,644
Resistant bulk	7,055,785	4,848,646	2,205,525	2.20	6,556,317	499,468
Susceptible bulk	7,393,507	5,080,134	2,311,724	2.20	6,829,194	564,313

SNPs, single nucleotide polymorphisms.

### 3.5 Indel identification, annotation, and positional distribution

It is believed that indel markers are a significant source of genetic markers that are commonly disseminated in genomes; however, they are not familiar as SNPs. The genetic alteration between individuals is determined as native changes comprising the replacement of small indels, which change small base pairs, and major alterations, in which large indels are reshuffled, resulting in copy number variations (Bansal and Libiger, 2011). Indels are the most regular fundamental variants that promote pathogenesis in diseases in animals, especially gene expression and function in humans (Montgomery et al., 2013). Current methods were used for the identification of indels, such as read splitting (Jiang et al., 2012), depth of coverage analysis (Abyzov et al., 2011), *de novo* assembly of unaligned reads (Li et al., 2013), and analysis of insert size variations.

GATK practices were used to compare the results of the BAM files. In addition, SNP detection and filtering were carried out using the GATK haplotype method. The filter conditions were used based on the parameters suggested by GATK (accessible online at https://software.broadinstitute.org/gatk/documentation/article.php?id=3225).

**Figure 3:**
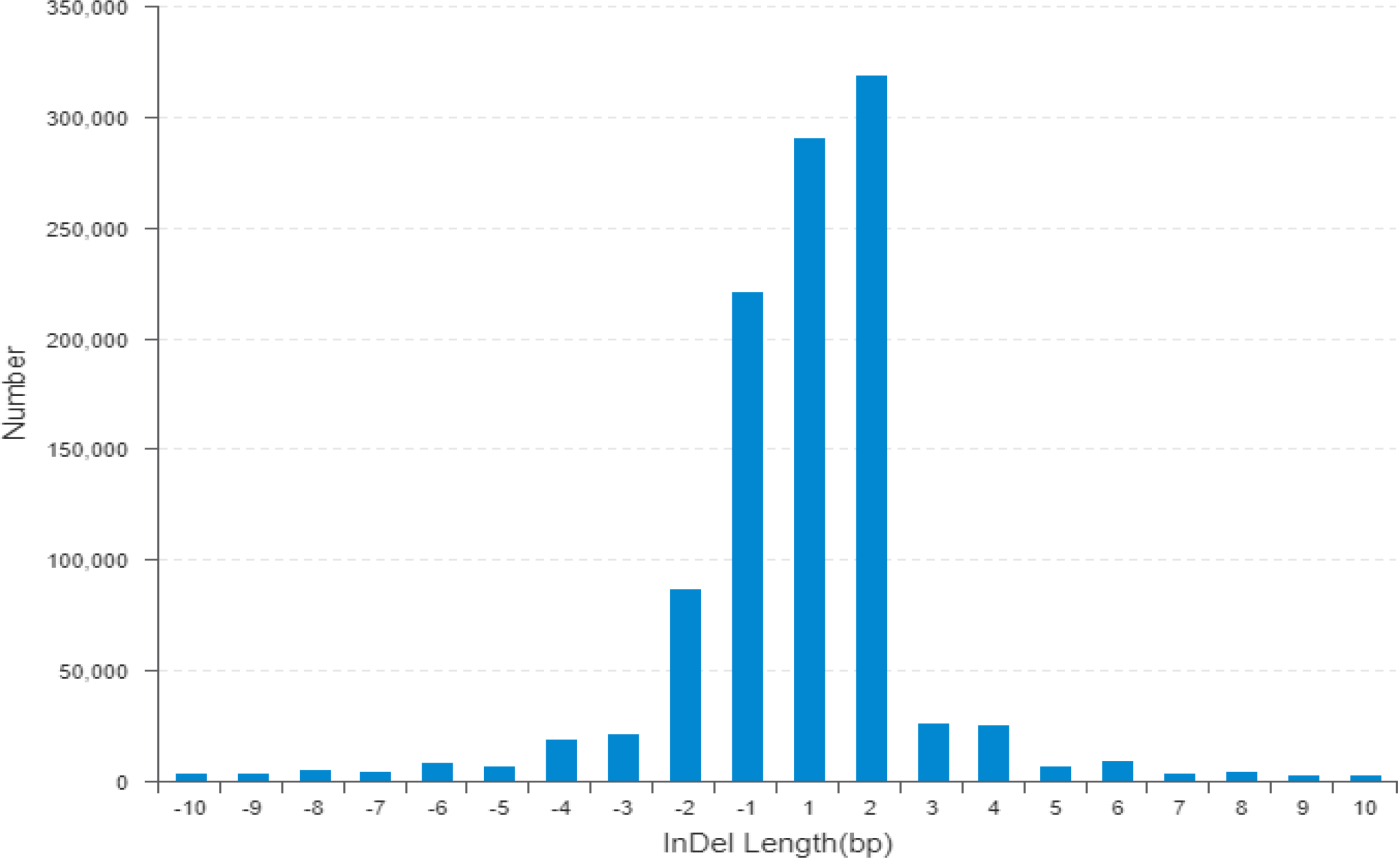
The horizontal line indicates the distribution of indel length (positive numbers show insertion length and negative numbers represent deletion length) and the longitudinal coordinate shows indel numbers by length. Indel, insertion/deletion.

The wild donor parent *G. darwinii* had a large insertion number (1,138,252), deletion number (879,688), heterozygosity number (427,622), and homozygosity number (1,590,318). The recurrent parent CCRI 12–4 had a low insertion number (341,608), deletion number (120,133), heterozygosity number (175,140), and homozygosity number (286,601) (**Figure 3**). The results showed that both bulks had high heterozygositynumbers (747,437 for the resistant bulk and 786,174 for the susceptible bulk) and low homozygosity numbers (284,477 for the resistant bulk and 301,048 for the susceptible bulk). The results indicated that BC_2_F_2_ bulks are more diverse than the recurrent parent (**Figure 3**; **Table S2**) (Hanada et al., 2007).

According to genetic variant annotation and the functional effect prediction toolbox (Snpeff), the influence of mutation sites on protein-coding has been divided into four levels, i.e., higher, moderate, lower, and modifier. The wild parent *G. darwinii* had larger numbers of indels with high functional mutations (7,665), moderate indels (3,202), low indels (3,425), and modifier indels that have no functions (2,030,744). In contrast, the recurrent parent CCRI 12–4 had smaller numbers of indels with high functional mutations (2,625), moderate indels (458), low indels (962), and modifier indels (474,073). The resistant bulk (4,234) and susceptible bulk (4,443) both have a larger number of indels with high mutational effects than the recurrent parent (**Supplementary Figure G2**).

### 3.6 Candidate gene prediction

To determine the candidate genomic regions that caused the difference between the two opposite poles by comparing their respective SNP/indel indices, we focused on the specific position of SNPs within the genome. The SNP/indel index is measured using a ratio of reads mapped to a specific position that represents a nucleotide substitution within the reference genome sequence. The SNP/indel index indicated the frequency of parent alleles in a population of bulked individuals. The *G. darwinii* genome was used as the reference genome for the resistant bulk and CCRI 12–4 for the susceptible bulk, with the resulting SNP index being one and its mean readings in the bulked population merely originating from CCRI 12–4. If the SNP index is zero, it indicates that the alleles are derived from *G. darwinii*. If the SNP index is 0.5, this indicates that both parents contributed equally. The ΔSNP index value was calculated through the substitution of a resistant pole index for the susceptible pole index.

Illumina high-throughput sequencing resulted in 347,192,723-bp and 110,919,876-bp reads for the resistant and susceptible bulks, respectively. The reads of bulk1 and bulk2 were aligned to the *G. darwinii* and *G. hirsutum* genomes, respectively, leading to the identification of 20,664,007 SNPs/indels. After the screening, 6,573 polymorphic markers were obtained after filtration in candidate regions. The SNP –index ( indices) of resistant and susceptible bulks and Δ(SNP-index) values of resistant and susceptible bulks were measured in a 1-Mb window using a 5-kb step with a 10 × sequencing depth (**Table S3**).

**Figure 4:**
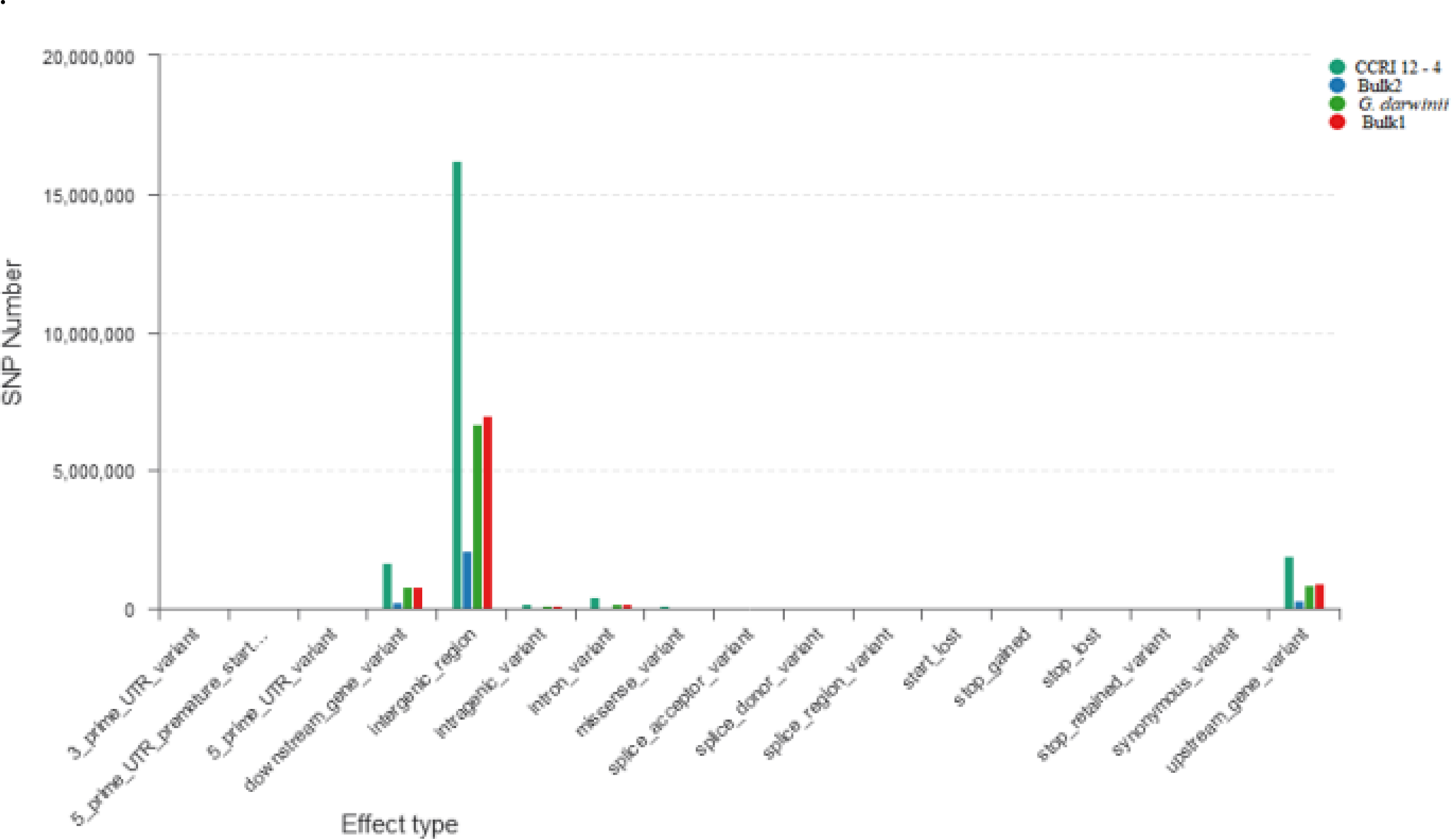
Horizontal coordinates indicate different classifications of the effect of SNPs on protein translation, and longitudinal coordinates represent the number of SNPs in the samples; different colors represent different samples. SNPs, single nucleotide polymorphisms.

**Figure 5:**
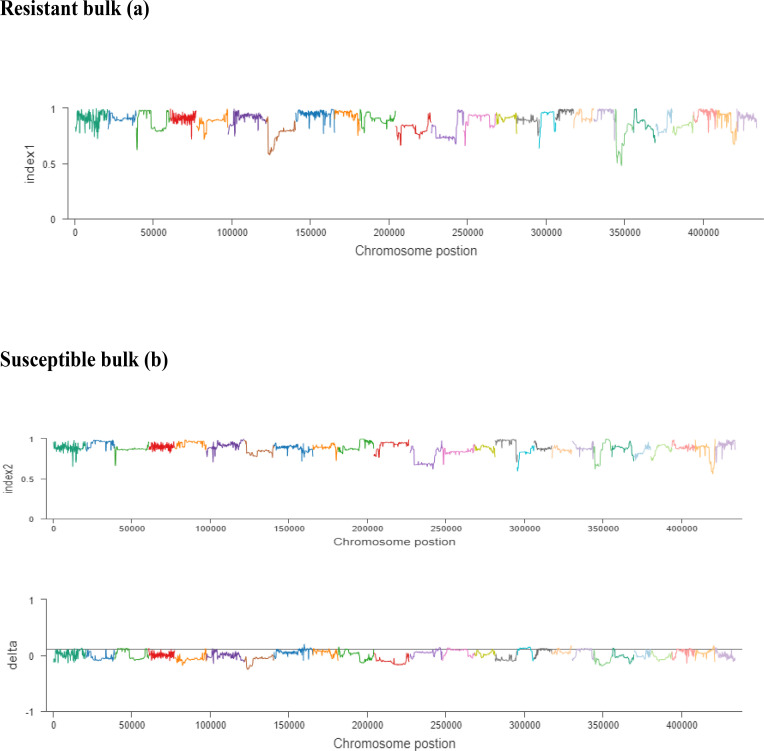
**(A, B).** The x-axis values are the specific physical location of each window on different chromosomes, the y-axis values are the index value corresponding to the position, and the threshold line is determined according to the threshold determination method. The resistant bulk is indicated by **(A)** and the susceptible bulk is indicated by **(B)**.

The ΔSNP index value is significantly different from 0 if a genomic region harbors a target gene. A total of 201 SNPs/indels were identified based on moderate and high mutation effects (**Table S4**). At the 95% significance level and higher, the Δ(SNP-index) value of six effective polymorphic SNPs was selected for the different chromosomes (**Table S5**). Four mutation regions were identified at the 99.9% significance level: one region was present on chromosome 18 (1.86 Mb) (positions 60,120,000–61,980,000) and had a Δ(SNP-index) ratio that was significantly different from zero. Two SNPs: one SNP was located in the stop–gain region and was a non-sense mutation, and the other was in the missense variant region and was associated with the same gene, *Gohir.D05G367800*, encoding the LRR receptor-like serine/threonine-specific protein kinase (**Figure 4**). Another two regions were lying on chromosome 25: Region 1 had a relative size of 1.06 Mb and was positioned between 57,795,000 and 58,850,000, and Region 2 was 1.94 Mb in size and was positioned between 58,890,000 and 60,825,000. In the first region, two SNPs were present, both found in the missense variant region; these SNPs were linked to the two genes *Gohir.D12G217800* and *Gohir.D12G224700*. It was predicted that *Gohir.D12G217800* was the E3 ubiquitin-protein ligase HOS1 and *Gohir.D12G224700* was the kinesin-like protein KIN-UA. The other region contained a single SNP that was present in the missense variant region associated with a linked gene, *Gohir.D12G239100*, which is involved in the production of B2 proteins and is thought to take part in stress responses and play a key role in plant growth and development. One SNP that was present in the missense variant region contained the *Gohir.A08G124300* gene on chromosome 8 and was positioned between 95,745,000–97,150,000. The *Gohir.A08G124300* gene is predicted to code for the serine/threonine-specific protein kinase WNK4. Six effective SNPs were linked to five candidate genes in four genomic regions (**Figure 5**).

**Figure 6:**
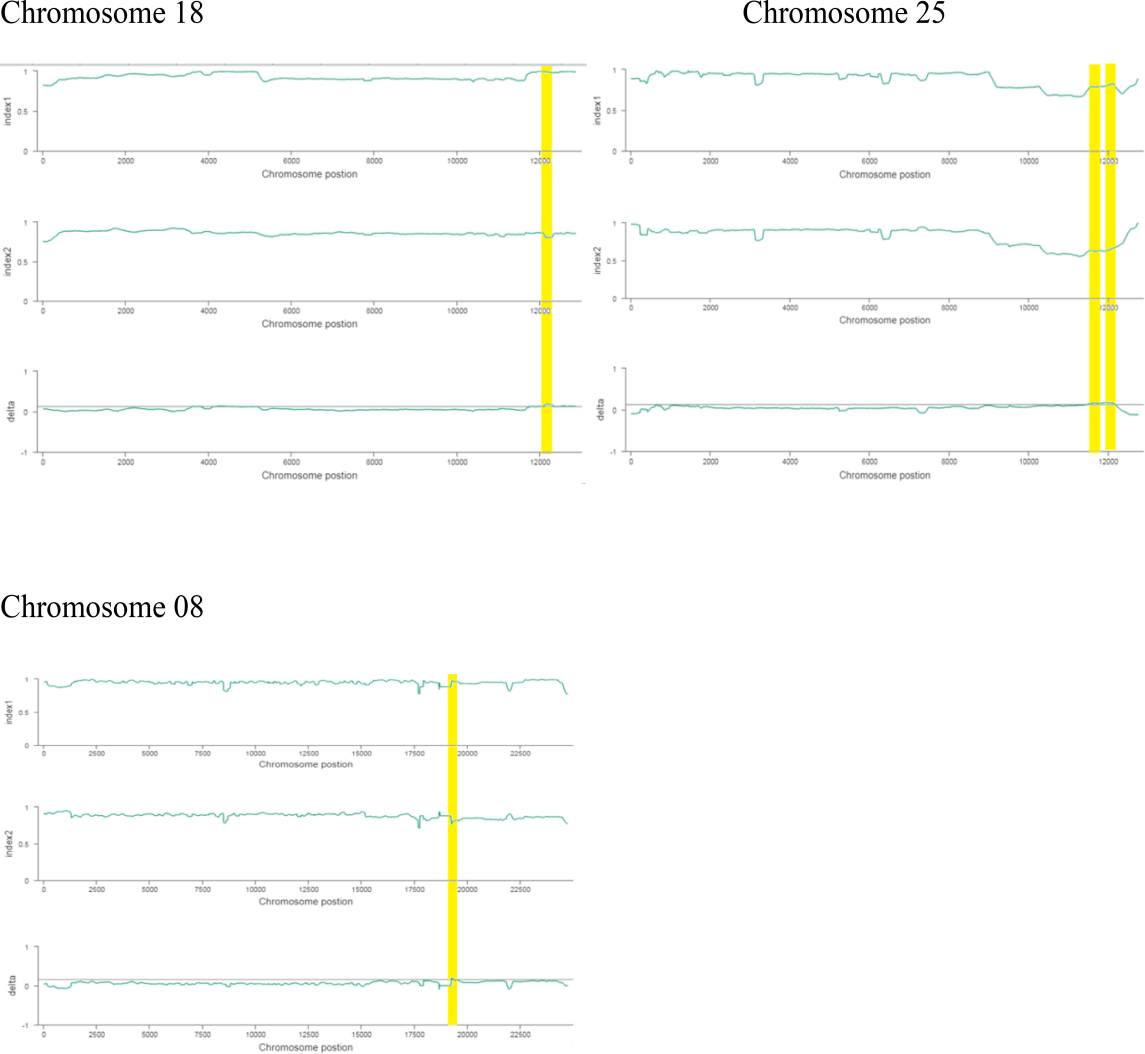
Index1 represents the resistant pole, index2 indicates the susceptible pole, and the delta index for the correlation analysis. The *x*-axis indicates the position of chromosomes, and the *y*-axis shows the SNPs index.

We noted the occurrence of five candidate salt tolerance genes on chromosomes 8, 18, and 25. The higher Δ(SNP-index) values remained in the 6098-bp stop–gain region, which was the non-sense mutation of *Gohir.D05G367800* on chromosome 18 and the 6020-bp missense mutation of *Gohir.D12G239100* on chromosome 25 (**Figure 6**). The *Gohir.D05G367800* gene also appeared promising based on the gene annotation results. The *Gohir.D05G367800* gene was predicted to be a leucine-rich repeat (LRR) receptor-like serine/threonine-specific protein kinase that contains the InterPro domain IPR000719. Moreover, the corresponding *A. thaliana* homolog for the LRR protein kinase family includes At3g47570. Protein BLAST alignment revealed that the sequence similarity between *Gohir.D05G367800* and At3g47570 was 43%. The LRR receptor-like serine/threonine-specific protein kinase plays a crucial role in the signaling cascade of the transmembrane receptor protein tyrosine kinase. The *Gohir.D05G367800* gene is the main possible candidate gene for salt stress tolerance in cotton plants. The *Gohir.D12G239100* gene, involved in the production of B2 proteins, is also linked to stress responses and play a vital role in the growth and development of plants (Singh and Khurana, 2016). According to publicly available transcript profiling data, the assumed *A. thaliana* ortholog At5g42050 of *Gohir.D12G239100* has the ability to tolerate different stresses, such as salinity, cold, and osmotic stress (Genevestigator: https://www.genevestigator.ethz.ch/).

### 3.7 Validation of candidate genes in *G. darwinii* with *G. hirsutum* as reference

**Figure 7:**
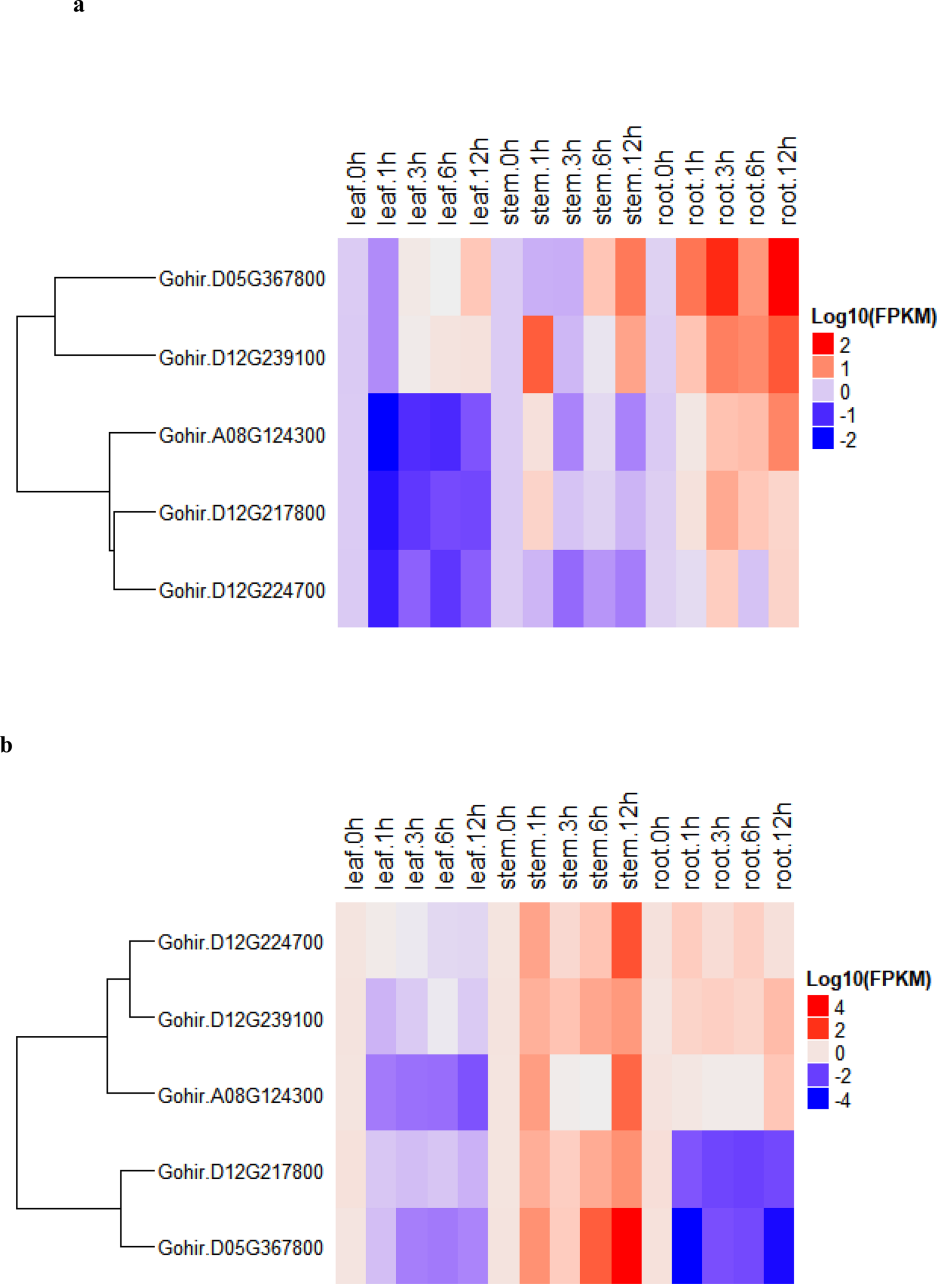
**(A, B). (A)** heatmap of *G. darwinii* and **(B)** *G. hirsutum*. Upregulated genes are represented by the red color, the blue color shows downregulated genes, while the white color shows no gene expression.

To determine the expression levels of five vital genes, these we studied their salt-stress responses in detail. We used the five candidate genes and designed primers for real-time RT-qPCR analysis (**Table S6**). The recurrent parent, CCRI12–4, and the donor parent, *G. darwinii* 5–7, were grown in controlled conditions in a greenhouse. RT-qPCR analysis was carried out for the two parents at 0-, 1-, 3-, 6-, and 12-hour time periods. Leaf, stem, and root samples were collected for RNA extraction. The results showed that five candidate genes had different expression levels under salt stress treatment, i.e., 200 mmol NaCl. The differing levels of expression allowed us to divide the genes into two groups in *G. hirsutum*. Group 1 genes were upregulated in all tissues except leaf tissue after salt stress, while higher expression was noted in stem and root tissue after 12 h of salt stress application. Group 2 had two genes that were more highly expressed in stem tissue, but downregulated in leaves and root tissues after salt stress treatments (**Figure 7A**).

In *G. darwinii*, expression patterns were also separated into two main groups, and the first group had three genes. The majority of group 1 genes were upregulated in all tissues of *G. darwinii* at 12 h of stress exposure, except in leaf tissue after 1 h, while in stem tissues, partially downregulated expression was observed after 1 and 3 h. Group 2 consisted of three genes, all of which were downregulated in leaf and stem tissues except for 1 h after salt treatment (**Figure 7B**). These three genes were upregulated in roots after 1 h and downregulated in root tissue after 3 h of salt treatment. These results show that these two vital genes are directly involved in the salt stress tolerance mechanism, particularly in root and stem tissues.

Furthermore, the two highly upregulated genes in *G. darwinii*, *Gohir.D05G367800*, and *Gohir.D12G239100*, had lower levels of gene expression in *G. hirsutum*, except *Gohir.D12G239100*. These two vital genes in *G. darwinii* had higher levels of gene expression in root tissue after 12 h of salt treatment, which showed the role they played in improving salt tolerance in developing roots. The results showed that certain significant alleles from the tolerant parent, *G. darwinii*, which was utilized as the donor parent for the creation of the BC_2_F_2_ mapping population, could have been introgressed into the population, potentially aiding in lessening the effect of salt stress in cotton plants. The majority of the genes in *G. darwinii* were significantly expressed in roots, whereas the majority of the genes in *G. hirsutum* were upregulated in stems. Different genes in both plants showed variable expression levels in different organs. *G. darwinii* demonstrated higher upregulation of a few genes in root tissues than *G. hirsutum*, indicating greater salt stress tolerance. We can hypothesize from these results that these two candidate genes are directly related to salt stress, particularly in roots.

## 4 Discussion

Salinity stress is the main abiotic stress that has a direct impact on cotton plants at the seedling stage and reduces cotton crop output. The mechanisms of salt stress tolerance have been the subject of many investigations, but the identification of salt tolerance genes has received less attention. However, salt-tolerant genes have been reported in wheat and barley (Genc et al., 2010). At present, upland cotton (*G. hirsutum*) varieties tolerant to salt stress are not grown in sufficient quantities to increase cotton production in salt stress conditions (Higbie et al., 2010). The transfer of salt tolerance from wild species to upland cotton is a feasible choice for the development of salt-tolerant cotton (Tiwari et al., 2013). The development of salt-tolerant cotton varieties is a rapid and effective way of reducing yield losses in areas where cotton is grown. The backcross population study is vital as compared with the inbred lines of the F_2_ population. In this study, we have successfully established a BC_2_F_2_ population that is a cross between the *G. hirsutum* and wild-type *G. darwinii* varieties. Both parents and the BC_2_F_2_ population were exposed to 150 mmol NaCl for the identification of salt stress-tolerance candidate genes.

In this study, we used hydroponic culture for the screening of salt-tolerant inbred lines rather than saline soil conditions because in a hydroponic system, the salt concentration is distributed equally to all plants, thus confirming that the genes identified are linked with the salt treatment. In a previous study, which reported on wheat, the results of salt tolerance measurements attained in water cultures were consistent with those of soil screening (Genc et al., 2007). Both parents and the BC_2_F_2_ population were exposed to 150 mmol NaCl at the seedling stage in controlled conditions in a greenhouse. Copious evidence has shown that plants at the seedling stage are most susceptible to salinity stress (Foolad and Jones, 1993). Under controlled conditions, all traits exhibited significant differences, except SFW and SDW. In addition, under controlled conditions, CCRI 12–4 produced more CHL than *G. Darwinii*. Enhanced levels of chlorophyll in the susceptible cultivar can be explained by the plant’s ability to increase photosynthetic activity in response to a sudden alteration in the environment that increases its survival. Our findings were consistent with previous findings indicating that higher levels of chlorophyll A and B and total chlorophyll contents were present in tolerant cultivars and were significantly lower in sensitive cultivars (Vadez et al., 2012). Highly significant differences were noted in all traits between the two parents under stressed conditions. Under salt stress conditions, all traits except RL, including CHL content, SFW, SDW, RFW, and RDW, were reduced. In a previous study, a 150 mmol NaCl application resulted in a significant decrease in growth for both a tolerant parent and a sensitive parent (Gossett et al., 1991). However, salt stress increased RL growth when *G. darwinii* was exposed to it. These findings support earlier research that reported on the improved growth of cotton under salt stress conditions (Tiwari and Stewart, 2008).

When exposed to salt stress, that is, when plants are treated with 100 mmol NaCl, the primary root length of the tolerant genotype plants is greater than that of the susceptible genotype plants (Leidi, 1994). The ability of *G. darwinii* to maintain a high photosynthetic rate despite limited shoot development, or the distinct reactions of its roots and shoots to phytohormones, may have produced root growth stimulation under salt stress (Youngner and Lunt, 1967). Tolerant and susceptible plants have been distinguished in some species based on root lengths, such as grasses (Liem et al., 1985) and sorghum (Azhar and McNeilly, 1988). Based on root length, *G. darwinii* has revealed some degree of salt tolerance, which can be transferred to upland cotton (*G. hirsutum*) to improve its tolerance against salt stress. The results indicate that CCRI12–4 showed tolerance to salt stress on above-ground traits, including CHL content, SFW, SDW, RFW, and RDW, whereas *G. darwinii* displayed salt tolerance on underground traits, such as RL.

The BSA analysis method was originally developed for the rapid identification of markers associated with particular gene/genomic regions in different crops, including cotton. This method provided an effective approach to the identification of genetic differences among bulk samples only within selected regions, but all these samples were heteromorphic and monomorphic for all genetic regions (Michelmore et al., 1991). The BSA technique, when combined with NGS, makes for a quick method for the identification of the main QTLs and genes associated with a particular trait (Singh et al., 2016).

In this study, two very distinctive phenotypic (resistant and susceptible) bulks were constructed; both bulks were sequenced, and clean data Q30 of 295.75 Gb was attained with 18,333,376 SNPs and 233,0631 indels. These SNPs/indels were used for the calculation of all SNP/indel genomic indexes. From sequence analysis, four candidate regions were identified for resistant and susceptible bulks. By BSA mapping analysis, four candidate regions were identified, one on chromosomes 8, 19, and 26, with five candidate genes responsible for salt stress tolerance. The total length of the candidate regions was 6.25 Mb. Similar findings were reported by Su et al. (2016), who identified five genomic regions on chromosome 2 with 0, 85, 13, 29, and 63 genes in maize (Su et al., 2016). The same type of research was also undertaken by Sun et al. (2018), where only two genes, *LOC_Os06g39740* and *LOC_Os06g39750*, were annotated with a function of the cold stress response, suggesting a key role in regulating cold tolerance in rice (Sun et al., 2018).

Technically, the two plants’ responses to salt stress are known, with *G. darwinii* being more salt-tolerant than the elite cultivar, *G. hirsutum*. Furthermore, *G. darwinii*, a wild allotetraploid species with an (AD)5 genome, is closely linked to *G. barbadense* but is substantially distinct from the farmed *G. hirsutum*, and it has several agronomically important features such as finer fiber fineness, drought/salt stress tolerance, and resistance to both *Fusarium* and *Verticillium* wilt (Liu F. et al., 2015). According to the results of RT-qPCR, based on the heatmaps, the five vital genes were highly expressed in *G. hirsutum* and expressed at lower levels in *G. darwinii* as per their log_10_ (FPKM, fragments per kilobase per million reads) range. In addition, *Gohir.D05G367800* and *Gohir.D12G239100* showed higher levels of expression in *G. darwinii* and lower levels in *G. hirsutum*, except *Gohir.D12G239100*. Even if root tissues are in direct contact with saline soil, an increase in solutes on root media, primarily ions, can induce a decrease in water intake from plant roots, causing a decline in root conductivity. As a result, plants take up the least amount of water, and if the transpiration rate exceeds the decrease in water absorption rate, the results can be reduced photosynthesis and growth rate (Zhou et al., 2013). Salinity influences both the reproductive and vegetative growth of plants, resulting in negative effects on plant organs such as the stem length, leaf length, root length, shoot length, fruit, fiber, or grain. Salt stress tends to suppress the first growth of the shoot as compared with root growth, and it can decrease blooming and enhance the chance of sterility (Wang et al., 2011).

The results confirmed that these salt-tolerant genes may have originated from a tolerant parent. These two candidate genes are salt-tolerant and could be useful for gene cloning, transformation, gene editing, and the generation of resistant cotton types in the future.

## 5 Conclusions

In the current study, the BC_2_F_2_ population developed from CRI12–4 × *G.darwinii* 5–7 was used for the identification of candidate genes under salt stress at the seedling stage in cotton plants. The Illumina HiSeq platform strategy with the NGS-based BSA technique was used as the gene mapping method. Previously, these techniques have most been used for gene mapping in different crop species. Total SNP/indels that were produced in this study in which Q30 ranged from 93.80 to 92.35, which indicated a high threshold for sequencing processes and error reduction to zero. Four genomic regions contained five candidate genes. Two genes showed higher expression levels under salt stress in *G. darwinii* than in *G. hirsutum,* and we recommend these as candidate genes. These are *Gohir.D05G367800*, an LRR receptor-like serine/threonine-specific protein kinase that has an important function in the transmembrane receptor protein tyrosine kinase signaling pathway, and *Gohir.D12G239100*, which is involved in the production of B2 proteins. Both of these genes are possibly involved in stress responses and play an key role in plant growth and developmental processes.

## Data availability statement

The datasets presented in this study can be found in the NCBI database with accession numbers: G. darwinii 5–7 and CCRI 12–4.

## Author contributions

MS, KW, and LF planned this research experiment. MS conducted the experiments, data collection, and bioinformatic analysis. MS, AD, and ZZ have contributed equally to the research experiments. MS, AD, and SU drafted and revised the manuscript. ZZ, XC, and YX reviewed the manuscript. All authors contributed to the writing of this study and approved the submitted version.

## References

[B1] Abdurakhmonov I. Kohel R. Yu J. Pepper A. Abdullaev A. Kushanov F.. (2008). Molecular diversity and association mapping of fiber quality traits in exotic g. hirsutum l. germplasm. Genomics 92 (6), 478–487. 18801424 10.1016/j.ygeno.2008.07.013

[B2] Abe A. Kosugi S. Yoshida K. Natsume S. Takagi H. Kanzaki H.. (2012). Genome sequencing reveals agronomically important loci in rice using MutMap. Nat. Biotechnol. 30 (2), 174. doi: 10.1038/nbt.2095 22267009

[B3] Abyzov A. Urban A. E. Snyder M. Gerstein M. (2011). CNVnator: an approach to discover, genotype, and characterize typical and atypical CNVs from family and population genome sequencing. Genome Res. 21 (6), 974–984. doi: 10.1101/gr.114876.110 21324876 PMC3106330

[B4] Azhar F. McNeilly T. (1988). The genetic basis of variation for salt tolerance in sorghum bicolor (L.) moench seedlings. Plant Breed. 101 (2), 114–121. doi: 10.1111/j.1439-0523.1988.tb00275.x

[B5] Bansal V. Libiger O. (2011). A probabilistic method for the detection and genotyping of small indels from population-scale sequence data. Bioinformatics 27 (15), 2047–2053. doi: 10.1093/bioinformatics/btr344 21653520 PMC3137221

[B6] Behera T. K. Staub J. E. Behera S. Delannay I. Y. Chen J. F. (2011). Marker-assisted backcross selection in an interspecific cucumis population broadens the genetic base of cucumber (Cucumis sativus l.). Euphytica 178, 261–272. doi: 10.1007/s10681-010-0315-8

[B7] Chen H. Khan M. K. R. Zhou Z. Wang X. Cai X. Ilyas M. K.. (2015). A high-density SSR genetic map constructed from a F2 population of gossypium hirsutum and gossypium darwinii. Gene 574 (2), 273–286. doi: 10.1016/j.gene.2015.08.022 26275937

[B8] Chen Z. Wang B. Dong X. Liu H. Ren L. Chen J.. (2014). An ultra-high density bin-map for rapid QTL mapping for tassel and ear architecture in a large f 2 maize population. BMC Genomics 15 (1), 433. doi: 10.1186/1471-2164-15-433 24898122 PMC4059873

[B9] Cingolani P. Platts A. Wang L. L. Coon M. Nguyen T. Wang L.. (2012). A program for annotating and predicting the effects of single nucleotide polymorphisms, SnpEff: SNPs in the genome of drosophila melanogaster strain w1118; iso-2; iso-3. Fly 6 (2), 80–92. doi: 10.4161/fly.19695 22728672 PMC3679285

[B10] Dabbert T. Gore M. A. (2014). Challenges and perspectives on improving heat and drought stress resilience in cotton. J. Cotton. Sci. 18 (3), 393–409.

[B11] Foolad M. Jones R. (1993). Mapping salt-tolerance genes in tomato (Lycopersicon esculentum) using trait-based marker analysis. Theor. Appl. Genet. 87 (1-2), 184–192. doi: 10.1007/BF00223763 24190211

[B12] Genc Y. Mcdonald G. K. Tester M. (2007). Reassessment of tissue na+ concentration as a criterion for salinity tolerance in bread wheat. Plant. Cell Environ. 30 (11), 1486–1498. doi: 10.1111/j.1365-3040.2007.01726.x 17897418

[B13] Genc Y. Oldach K. Verbyla A. P. Lott G. Hassan M. Tester M.. (2010). Sodium exclusion QTL associated with improved seedling growth in bread wheat under salinity stress. Theor. Appl. Genet. 121 (5), 877–894. doi: 10.1007/s00122-010-1357-y 20490443

[B14] Gossett D. Millhollon E. Caldwell W. Mundy S. (1991). “Isozyme variation among salt tolerant and salt sensitive varieties of cotton,” in Proceedings-Beltwide Cotton Conferences (USA). National Cotton Council Memphis, TN.1036-1039.

[B15] Hamwieh A. Imtiaz M. Malhotra R. (2013). Multi-environment QTL analyses for drought-related traits in a recombinant inbred population of chickpea (Cicer arientinum l.). Theor. Appl. Genet. 126 (4), 1025–1038. doi: 10.1007/s00122-012-2034-0 23283512

[B16] Han Y. Lv P. Hou S. Li S. Ji G. Ma X.. (2015). Combining next generation sequencing with bulked segregant analysis to fine map a stem moisture locus in sorghum (Sorghum bicolor l. moench). PloS One 10 (5), e0127065. doi: 10.1371/journal.pone.0127065 25984727 PMC4436200

[B17] Hanada K. Zhang X. Borevitz J. O. Li W.-H. Shiu S.-H. (2007). A large number of novel coding small open reading frames in the intergenic regions of the arabidopsis thaliana genome are transcribed and/or under purifying selection. Genome Res. 17 (5), 632–640. doi: 10.1101/gr.5836207 17395691 PMC1855179

[B18] He C. Yan J. Shen G. Fu L. Holaday A. S. Auld D.. (2005). Expression of an arabidopsis vacuolar sodium/proton antiporter gene in cotton improves photosynthetic performance under salt conditions and increases fiber yield in the field. Plant Cell Physiol. 46 (11), 1848–1854. doi: 10.1093/pcp/pci201 16179357

[B19] Higbie S. M. Wang F. Stewart J. M. Sterling T. M. Lindemann W. C. Hughs E.. (2010). Physiological response to salt (NaCl) stress in selected cultivated tetraploid cottons. Int. J. Agron. 2010. Article ID 643475, 12 pages doi: 10.1155/2010/643475

[B20] Hoagland D. R. Arnon D. I. (1950). The water-culture method for growing plants without soil. circular. California. Agric. Experiment. Station 347 (2nd edit).

[B21] Hospital F. (2005). Selection in backcross programmes. Philos. Trans. R. Soc. B.: Biol. Sci. 360 (1459), 1503–1511. doi: 10.1098/rstb.2005.1670 PMC1569518 16048792

[B22] Jiang Y. Wang Y. Brudno M. (2012). PRISM: pair-read informed split-read mapping for base-pair level detection of insertion, deletion and structural variants. Bioinformatics 28 (20), 2576–2583. doi: 10.1093/bioinformatics/bts484 22851530

[B23] Kirungu J. Deng Y. Cai X. Magwanga R. Zhou Z. Wang X.. (2018). Simple sequence repeat (SSR) genetic linkage map of d genome diploid cotton derived from an interspecific cross between gossypium davidsonii and gossypium klotzschianum. Int. J. Mol. Sci. 19 (1), 204. doi: 10.3390/ijms19010204 29324636 PMC5796153

[B24] Klein H. Xiao Y. Conklin P. A. Govindarajulu R. Kelly J. A. Scanlon M. J.. (2018). Bulked-segregant analysis coupled to whole genome sequencing (BSA-seq) for rapid gene cloning in maize. G3.: Genes. Genomes. Genet. 8 (11), 3583–3592. doi: 10.1534/g3.118.200499 PMC6222591 30194092

[B25] Leidi E.O. (1994). Genotypic variation of cotton in response to stress by NaCl or PEG. In: Peeters M.C. (ed) Cotton biotechnology., REUR technical series 32FAO, Rome, 67–73.

[B26] Li H. Durbin R. (2009). Fast and accurate short read alignment with burrows–wheeler transform. bioinformatics 25 (14), 1754–1760. doi: 10.1093/bioinformatics/btp324 19451168 PMC2705234

[B27] Li F. Fan G. Lu C. Xiao G. Zou C. Kohel R. J.. (2015). Genome sequence of cultivated upland cotton (Gossypium hirsutum TM-1) provides insights into genome evolution. Nat. Biotechnol. 33 (5), 524. doi: 10.1038/nbt.3208 25893780

[B28] Li F. Fan G. Wang K. Sun F. Yuan Y. Song G.. (2014). Genome sequence of the cultivated cotton gossypium arboreum. Nat. Genet. 46 (6), 567. doi: 10.1038/ng.2987 24836287

[B29] Li H. Handsaker B. Wysoker A. Fennell T. Ruan J. Homer N.. (2009). The sequence alignment/map format and SAMtools. Bioinformatics 25 (16), 2078–2079. doi: 10.1093/bioinformatics/btp352 19505943 PMC2723002

[B30] Li S. Li R. Li H. Lu J. Li Y. Bolund L.. (2013). SOAPindel: efficient identification of indels from short paired reads. Genome Res. 23 (1), 195–200. doi: 10.1101/gr.132480.111 22972939 PMC3530679

[B31] Liem A. Hendriks A. Kraal H. Loenen M. (1985). Effects of de-icing salt on roadside grasses and herbs. Plant Soil 84 (3), 299–310. doi: 10.1007/BF02275470

[B32] Liu X. Zhao B. Zheng H.-J. Hu Y. Lu G. Yang C.-Q.. (2015). Gossypium barbadense genome sequence provides insight into the evolution of extra-long staple fiber and specialized metabolites. Sci. Rep. 5, 14139. doi: 10.1038/srep14139 26420475 PMC4588572

[B33] Liu F. Zhou Z.L. Wang C.Y. Wang Y.H. Cai X.-Y. Wang X.X.. (2015). Collinearity analysis of allotetraploid gossypium tomentosum and gossypium darwinii. Genet Mol Res 15 (3). doi: 10.1101/031104 27525913

[B34] Livaja M. Wang Y. Wieckhorst S. Haseneyer G. Seidel M. Hahn V.. (2013). BSTA: a targeted approach combines bulked segregant analysis with next-generation sequencing and de novo transcriptome assembly for SNP discovery in sunflower. BMC Genomics 14 (1), 628. doi: 10.1186/1471-2164-14-628 24330545 PMC3848877

[B35] Lu Y. Wu X. Yao M. Zhang J. Liu W. Yang X.. (2015). Genetic mapping of a putative agropyron cristatum-derived powdery mildew resistance gene by a combination of bulked segregant analysis and single nucleotide polymorphism array. Mol. Breed. 35 (3), 96. doi: 10.1007/s11032-015-0292-7

[B36] Magwanga R. O. Lu P. Kirungu J. N. Lu H. Wang X. Cai X.. (2018). Characterization of the late embryogenesis abundant (LEA) proteins family and their role in drought stress tolerance in upland cotton. BMC Genet. 19 (1), 1–31. doi: 10.1186/s12863-017-0596-1 29334890 PMC5769447

[B37] McKenna A. Hanna M. Banks E. Sivachenko A. Cibulskis K. Kernytsky A.. (2010). The genome analysis toolkit: a MapReduce framework for analyzing next-generation DNA sequencing data. Genome Res. 20 (9), 1297–1303. doi: 10.1101/gr.107524.110 20644199 PMC2928508

[B38] Michelmore R. W. Paran I. Kesseli R. (1991). Identification of markers linked to disease-resistance genes by bulked segregant analysis: a rapid method to detect markers in specific genomic regions by using segregating populations. Proc. Natl. Acad. Sci. 88 (21), 9828–9832. doi: 10.1073/pnas.88.21.9828 1682921 PMC52814

[B39] Montgomery S. B. Goode D. L. Kvikstad E. Albers C. A. Zhang Z. D. Mu X. J.. (2013). The origin, evolution, and functional impact of short insertion–deletion variants identified in 179 human genomes. Genome Res. 23 (5), 749–761. doi: 10.1101/gr.148718.112 23478400 PMC3638132

[B40] Oluoch G. Zheng J. Wang X. Khan M. K. R. Zhou Z. Cai X.. (2016). QTL mapping for salt tolerance at seedling stage in the interspecific cross of gossypium tomentosum with gossypium hirsutum. Euphytica 209 (1), 223–235. doi: 10.1007/s10681-016-1674-6

[B41] Peng Z. He S. Gong W. Sun J. Pan Z. Xu F.. (2014). Comprehensive analysis of differentially expressed genes and transcriptional regulation induced by salt stress in two contrasting cotton genotypes. BMC Genomics 15 (1), 760. doi: 10.1186/1471-2164-15-760 25189468 PMC4169805

[B42] Reinhardt D. Rost T. (1995). Primary and lateral root development of dark-and light-grown cotton seedlings under salinity stress. Botanica Acta 108 (5), 457–465. doi: 10.1111/j.1438-8677.1995.tb00521.x

[B43] Renny-Byfield S. Page J. T. Udall J. A. Sanders W. S. Peterson D. G. Arick M. A.. (2016). Independent domestication of two old world cotton species. Genome Biol. Evol. 8 (6), 1940–1947. doi: 10.1093/gbe/evw129 27289095 PMC4943200

[B44] Schneeberger K. Ossowski S. Lanz C. Juul T. Petersen A. H. Nielsen K. L.. (2009). SHOREmap: simultaneous mapping and mutation identification by deep sequencing. Nat. Methods 6 (8), 550. doi: 10.1038/nmeth0809-550 19644454

[B45] Shehzad M. Zhou Z. Ditta A. Cai X. Khan M. Xu Y.. (2019). Genome-wide mining and identification of protein kinase gene family impacts salinity stress tolerance in highly dense genetic map developed from interspecific cross between g. hirsutum l. and g. darwinii g. watt. Agronomy 9 (9), 560. doi: 10.3390/agronomy9090560

[B46] Singh V. K. Khan A. W. Saxena R. K. Kumar V. Kale S. M. Sinha P.. (2016). Next-generation sequencing for identification of candidate genes for fusarium wilt and sterility mosaic disease in pigeonpea (C ajanus cajan). Plant Biotechnol. J. 14 (5), 1183–1194. doi: 10.1111/pbi.12470 26397045 PMC5054876

[B47] Singh A. Khurana P. (2016). Molecular and functional characterization of a wheat B2 protein imparting adverse temperature tolerance and influencing plant growth. Front. Plant Sci. 7, 642. doi: 10.3389/fpls.2016.00642 27242843 PMC4861841

[B48] Su A. Song W. Xing J. Zhao Y. Zhang R. Li C.. (2016). Identification of genes potentially associated with the fertility instability of s-type cytoplasmic male sterility in maize via bulked segregant RNA-seq. PloS One 11 (9), e0163489. doi: 10.1371/journal.pone.0163489 27669430 PMC5036866

[B49] Sun J. Yang L. Wang J. Liu H. Zheng H. Xie D.. (2018). Identification of a cold-tolerant locus in rice (Oryza sativa l.) using bulked segregant analysis with a next-generation sequencing strategy. Rice 11 (1), 24. 29671148 10.1186/s12284-018-0218-1 PMC5906412

[B50] Takagi H. Abe A. Yoshida K. Kosugi S. Natsume S. Mitsuoka C.. (2013). QTL-seq: rapid mapping of quantitative trait loci in rice by whole genome resequencing of DNA from two bulked populations. Plant J. 74 (1), 174–183. doi: 10.1111/tpj.12105 23289725

[B51] Takeda S. Matsuoka M. (2008). Genetic approaches to crop improvement: responding to environmental and population changes. Nat. Rev. Genet. 9 (6), 444. doi: 10.1038/nrg2342 18475268

[B52] Tiwari R. S. Stewart J. M. (2008). Effect of salt on several genotypes of gossypium. Summaries. Arkansas. Cotton. Res. 2. 573:34–36.

[B53] Tiwari R. S. Picchioni G. A. Steiner R. L. Jones D. C. Hughs S. E. Zhang J. (2013). Genetic variation in salt tolerance at the seedling stage in an interspecific backcross inbred line population of cultivated tetraploid cotton. Euphytica 194, 1–11.

[B54] Trick M. Adamski N. M. Mugford S. G. Jiang C.-C. Febrer M. Uauy C. (2012). Combining SNP discovery from next-generation sequencing data with bulked segregant analysis (BSA) to fine-map genes in polyploid wheat. BMC Plant Biol. 12 (1), 14. doi: 10.1186/1471-2229-12-14 22280551 PMC3296661

[B55] Uma M. Patil B. (1996). Inter-species variation in the performance of cotton under soil salinity stress. Karnatak. J. Agric. Res. 9 (1), 73–77. Available at: https://eurekamag.com/research/002/876/002876051.php

[B56] Vadez V. Krishnamurthy L. Thudi M. Anuradha C. Colmer T. D. Turner N. C.. (2012). Assessment of ICCV 2× JG 62 chickpea progenies shows sensitivity of reproduction to salt stress and reveals QTL for seed yield and yield components. Mol. Breed. 30 (1), 9–21. doi: 10.1007/s11032-011-9594-6

[B57] Varshney R. K. Terauchi R. McCouch S. R. (2014). Harvesting the promising fruits of genomics: applying genome sequencing technologies to crop breeding. PloS Biol. 12 (6), e1001883. doi: 10.1371/journal.pbio.1001883 24914810 PMC4051599

[B58] Wambugu P. Ndjiondjop M. N. Furtado A. Henry R. (2018). Sequencing of bulks of segregants allows dissection of genetic control of amylose content in rice. Plant Biotechnol. J. 16 (1), 100–110. doi: 10.1111/pbi.12752 28499072 PMC5785344

[B59] Wang K. Wang Z. Li F. Ye W. Wang J. Song G.. (2012). The draft genome of a diploid cotton gossypium raimondii. Nat. Genet. 44 (10), 1098. doi: 10.1038/ng.2371 22922876

[B60] Wang W. Wang R. Yuan Y. Du N. Guo W. (2011). Effects of salt and water stress on plant biomass and photosynthetic characteristics of tamarisk (Tamarix chinensis lour.) seedlings. Afr. J. Biotechnol. 10 (78), 17981–17989. doi: 10.5897/AJB11.1864

[B61] Wang X. Lu X. Malik W. A. Chen X. Wang J. Wang D. (2020). Differentially expressed bZIP transcription factors confer multi-tolerances in Gossypium hirsutum L. Int. J. Biol. Macromol. 146, 569–578. 31923491 10.1016/j.ijbiomac.2020.01.013

[B62] Whipple C. J. Kebrom T. H. Weber A. L. Yang F. Hall D. Meeley R.. (2011). Grassy tillers1 promotes apical dominance in maize and responds to shade signals in the grasses. Proc. Natl. Acad. Sci. 108 (33), E506–E512. doi: 10.1073/pnas.1102819108 21808030 PMC3158142

[B63] Youngner V. Lunt O. (1967). Salinity effects on roots and tops of bermuda grass. Grass. Forage. Sci. 22 (4), 257–259. doi: 10.1111/j.1365-2494.1967.tb00536.x

[B64] Zhang J. Percy R. G. McCarty J. C. (2014). Introgression genetics and breeding between upland and pima cotton: a review. Euphytica 198 (1), 1–12. doi: 10.1007/s10681-014-1094-4

[B65] Zhou Z. Sun L. Zhao Y. An L. Yan A. Meng X. Gan Y. (2013). Z inc F inger P rotein 6 (ZFP 6) regulates trichome initiation by integrating gibberellin and cytokinin signaling in A rabidopsis thaliana. New Phytologist 198(3), 699–708. 23506479 10.1111/nph.12211

[B66] Zhu J.-K. (2001). Cell signaling under salt, water and cold stresses. Curr. Opin. Plant Biol. 4 (5), 401–406. doi: 10.1016/S1369-5266(00)00192-8 11597497

